# Supplementary material for: Universal Approach to Integrating Reduced Graphene Oxide into Polymer Electronics
Source: Polymers (Basel). 2023 Dec 5;15(24):4622. doi: 10.3390/polym15244622 (PMC10747855; doi:10.3390/polym15244622)
Supplement: Supplementary file 1 [file polymers-15-04622-s001.zip › polymers-2723618-supplementary.pdf]

Supplementary Materials

Universal Approach to Integrating Reduced Graphene Oxide into Polymer Electronics

Elena Abyzova [1], Ilya Petrov [1], Ilya Bril′ [1], Dmitry Cheshev [1], Alexey Ivanov [1], Maxim Khomenko [2], Andrey Averkiev [1], Maxim Fatkulin [1], Dmitry Kogolev [1], Evgeniy Bolbasov [1], Aleksandar Matkovic [3,*], Jin-Ju Chen [4], Raul D. Rodriguez [1,*] and Evgeniya Sheremet [1]

[1] Research School of Chemistry & Applied Biomedical Sciences, Tomsk Polytechnic University, Lenina Ave, 30, 634050 Tomsk, Russia; ellaijiah@gmail.com (I.B.); kogolev@tpu.ru (D.K.)
[2] ILIT RAS–Branch of the FSRC "Crystallography and Photonics" RAS, 140700 Shatura, Russia
[3] Department Physics, Mechanics and Electrical Engineering, Montanuniversität Leoben, Franz Josef Strasse 18, 8700 Leoben, Austria
[4] School of Materials and Energy, University of Electronic Science and Technology of China, Chengdu 610054, China; jinjuchen@uestc.edu.cn
* Correspondence: aleksandar.matkovic@unileoben.ac.at (A.M.); raul@tpu.ru (R.D.R.)

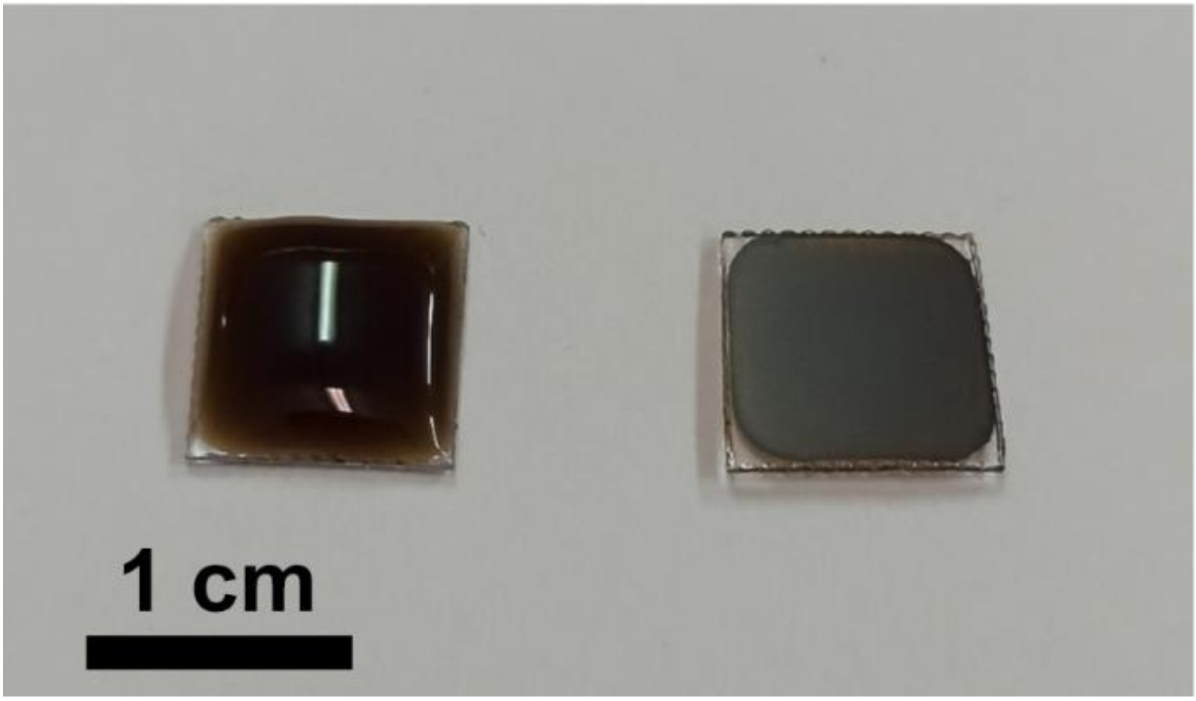

Figure S1. GO on PET (left) and dry GO film on PET (right)

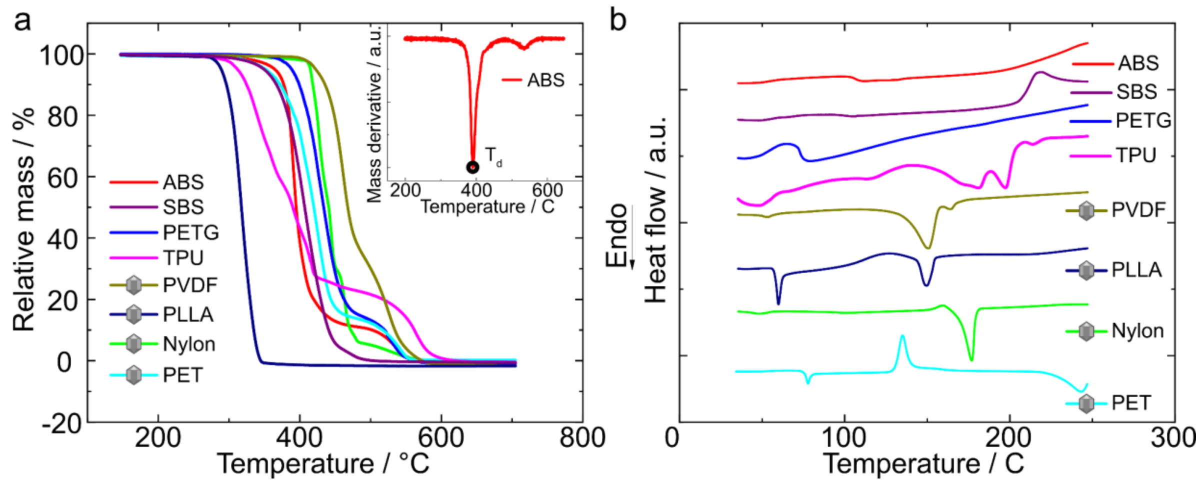

Figure S2. (a) TGA and (b) DSC curves for the set of polymers. Combustion temperature was determined using the 1st derivative of the mass curve as displayed in the inset of the TGA plot. Polymers with high crystallinity are marked with a symbol

Table S1. Thermal properties of polymers

Polymer	T_g / °C	T_m / °C	T_d / °C	Crystallinity	Fragility	ΔH_f / J/g
ABS	107	N/A, amorphous	390	Low	LOW [1]	N/A, amorphous
Nylon	96	177	426	High	-	58.9
PETG	73	N/A, amorphous	425	Low	High 126 [2]	N/A, amorphous
PET	76	242	424	High	-	N/A, out of range
TPU	N/A (~ -40)[3]	180	417	semi	High [4]	11.2
PVDF	N/A (< -35)[5]	151	461	High	-	14.1
PLLA	60	150	316	High	-	7.9
SBS	N/A (-55)[6]	N/A, amorphous	405	Low	Very high 292.4 [7]	N/A, amorphous

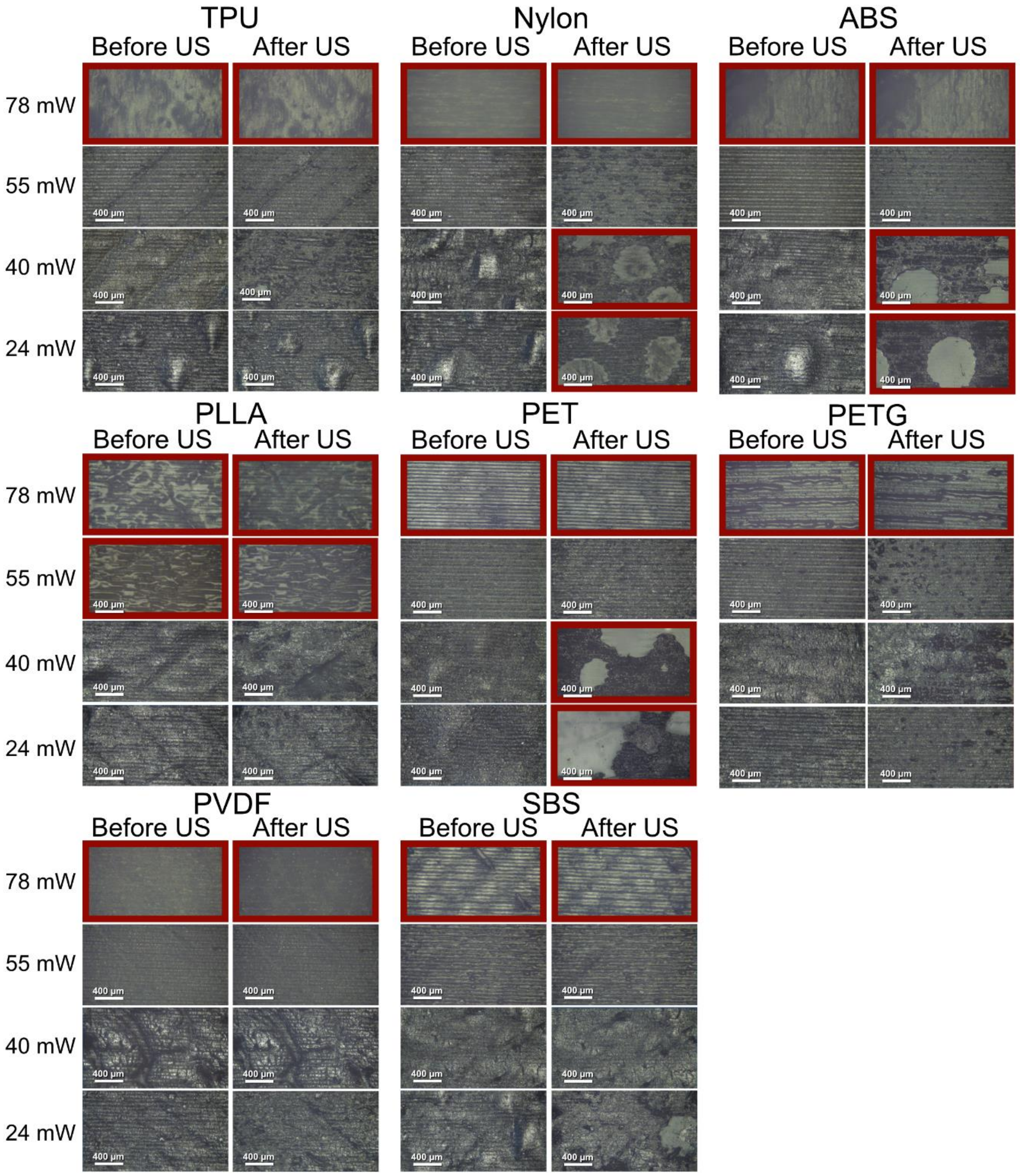

Figure S3. Optical images of rGO/polymer samples processed at different laser power before and after ultrasonication. A red frame is used to indicate non-conductive samples.

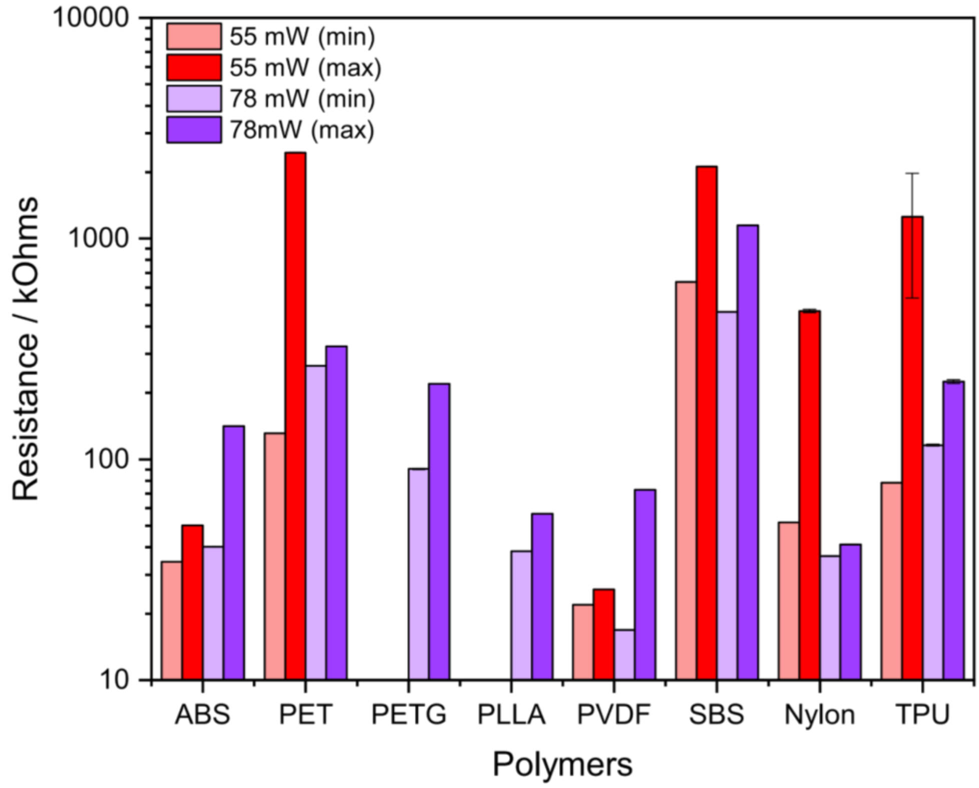

Figure S4. Resistance of a single rGO/polymer composite lines

Table S2. All polymer parameters used in the COMSOL model

Polymer	Density, kg/m^3	Thermal conductivity, W/m*K	Specific heat capacity, J/Kg*°C
PET	1397[8]	0.3[9]	1200[10]
PETG	1300[11]	0.21[12]	1200[13]
ABS	1050[14,15]	0.33[16,17]	1300[16]
PLA	1250[14,18]	0.183[19]	1800[18]
Nylon	1150[20,21]	0.25[22]	1667[23]
TPU	1135[24]	0.15[25]	1210[25]
PVDF	1780[26,27]	0.181[28]	1250[27,29]
SBS	1023[30,31]	0.191[32]	1540[32]

FEM simulation model

The maximum polymer temperature is almost (99.9%) equal to the maximum GO temperature since the GO film is much thinner in comparison with the polymer substrate. The physical parameters for GO film were taken from these works[33–39].

FEM Modelling details

Commercial software COMSOL Multiphysics was employed to simulate the laser heating of GO/Polymers composites. The laser heating was modeled as a power source with a rectangular shape measuring 30x100 μm and a top-hat power distribution, using the Time-Dependent Heat Transfer in the Solids module. The samples were set as two layers: the polymer layer and the GO layer on top. The GO film had dimensions of 1.5x1.5 mm with a thickness of 670 nm, while all polymers were modeled with consistent dimensions, measuring 1.5x1.5 mm and having a thickness of 0.65 mm. Due to its thinness, the GO layer is represented as a single mesh layer, in contrast to the underlying polymer which is depicted with multiple mesh layers. For precise simulation, we applied extremely fine mesh settings to the top GO layer in the laser heating source region, with a minimum element size of 0.3 μm. The remainder of the model was set to extra fine mesh with a minimum element size of 2.3 μm, see the meshed model in Figure S5d. It is important to note that, given the thinness of the GO layer, the temperature induced by the laser on both sides of the GO film is nearly identical, minimizing the impact of the number of mesh layers in the GO itself. For the heating of all the samples, the following power values were utilized: 24, 40, 55, and 78 mW. The time of the laser heating was set to 0.25 seconds. These parameter selections were made to represent the experimental conditions.

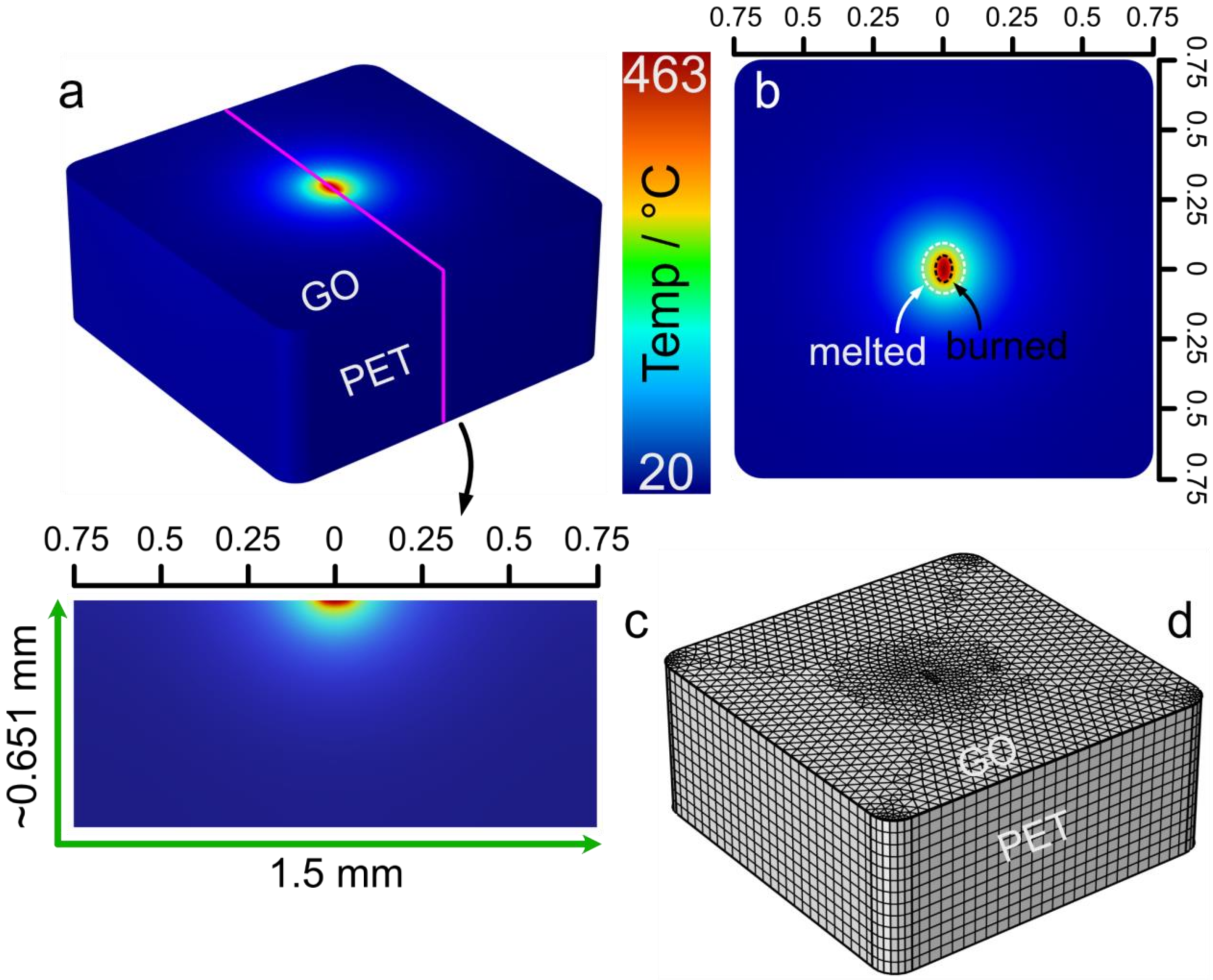

Figure S5. COMSOL model of the GO/Polymer composite after 0.25 seconds of the laser heating at 78 mW power. (a) Side view, (b) Top view, (c) Cross-section from the region indicated in (a), (d) Meshed model.

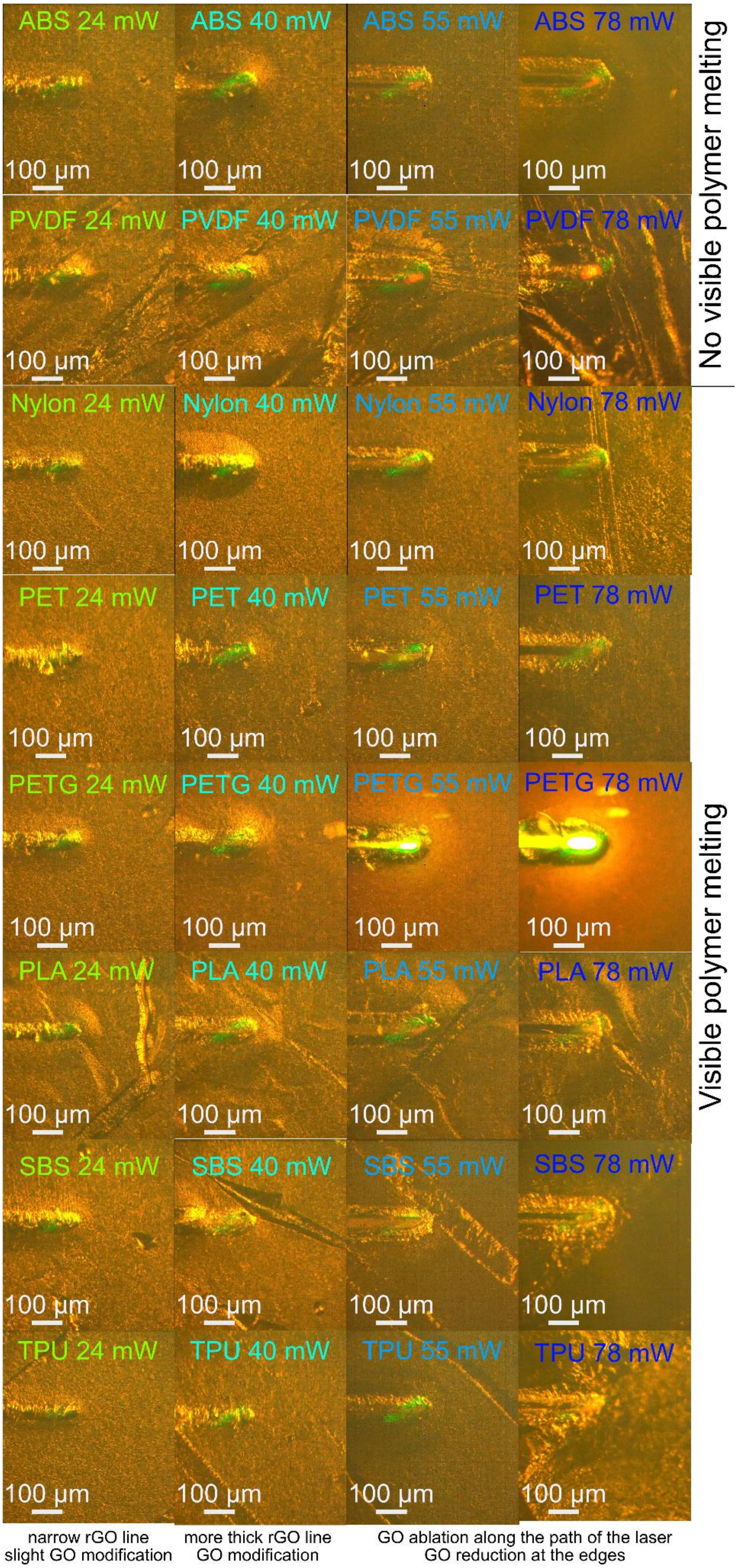
ABS 24 mW
ABS 40 mW
ABS 55 mW
ABS 78 mW
PVDF 24 mW
PVDF 40 mW
PVDF 55 mW
PVDF 78 mW
Nylon 24 mW
Nylon 40 mW
Nylon 55 mW
Nylon 78 mW
PET 24 mW
PET 40 mW
PET 55 mW
PET 78 mW
PETG 24 mW
PETG 40 mW
PETG 55 mW
PETG 78 mW
PLA 24 mW
PLA 40 mW
PLA 55 mW
PLA 78 mW
SBS 24 mW
SBS 40 mW
SBS 55 mW
SBS 78 mW
TPU 24 mW
TPU 40 mW
TPU 55 mW
TPU 78 mW
100 µm
No visible polymer melting
Visible polymer melting
narrow rGO line
slight GO modification
more thick rGO line
GO modification
GO ablation along the path of the laser
GO reduction at the edges

Figure S6. A frame high-speed camera recording during laser processing of GO/polymer with laser power 24, 40, 55, and 78 mW.

Table S3. The width of the modified area of materials for powers of 24, 40, 55, and 78 mW before and after 1-minute treatment in the ultrasonic bath.

		Nylon	PVDF	SBS	TPU	PETG	PET	ABS	PLLA
24 mW	Before US	100	125	150	110	150	170	220	240
	After US	75	140	165	95	160	165	140	255
40 mW	Before US	330	290	390	300	360	300	300	530
	After US	140	200	240	145	260	230	210	390
55 mW	Before US	330	185	200	200	340	165	585	330
	After US	165	240	190	200	330	325	250	470
78 mW	Before US	300	160	200	275	250	150	260	290
	After US	200	325	210	210	220	400	240	570

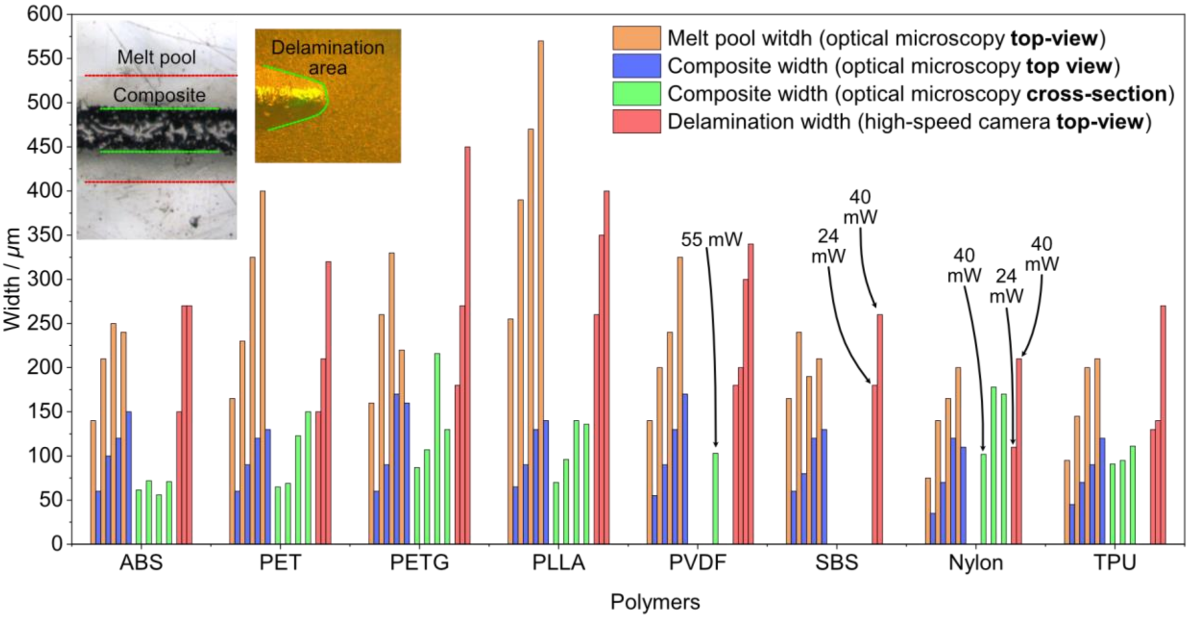

Figure S7. Histogram representing width of different areas of lines taken from high-speed

recordings and cross-section images. Each column represents a certain power from left to right (24, 40, 55, and 78 mW).

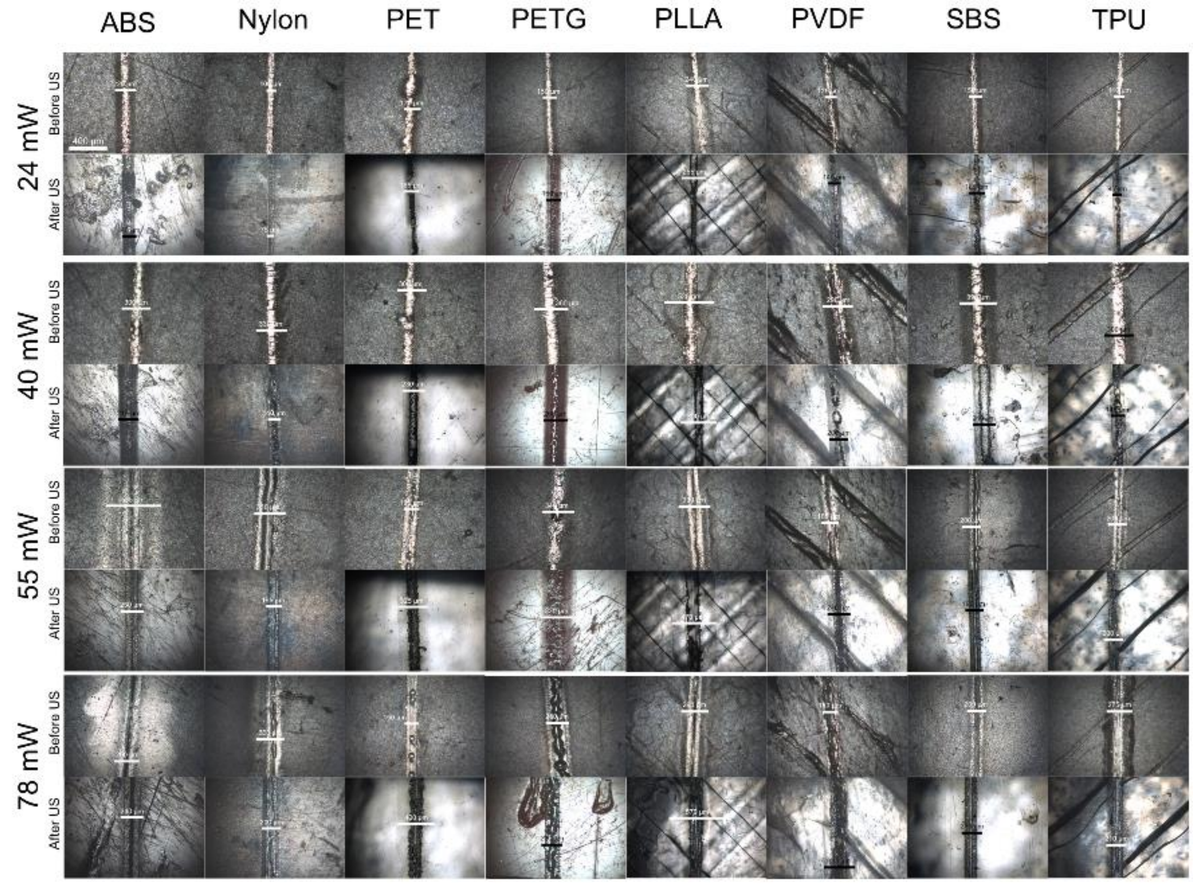

Figure S8. Optical images of laser-induced lines at a power of 24, 40, 55, and 78 mW on polymers before and after 1-minute treatment in an ultrasonic bath.

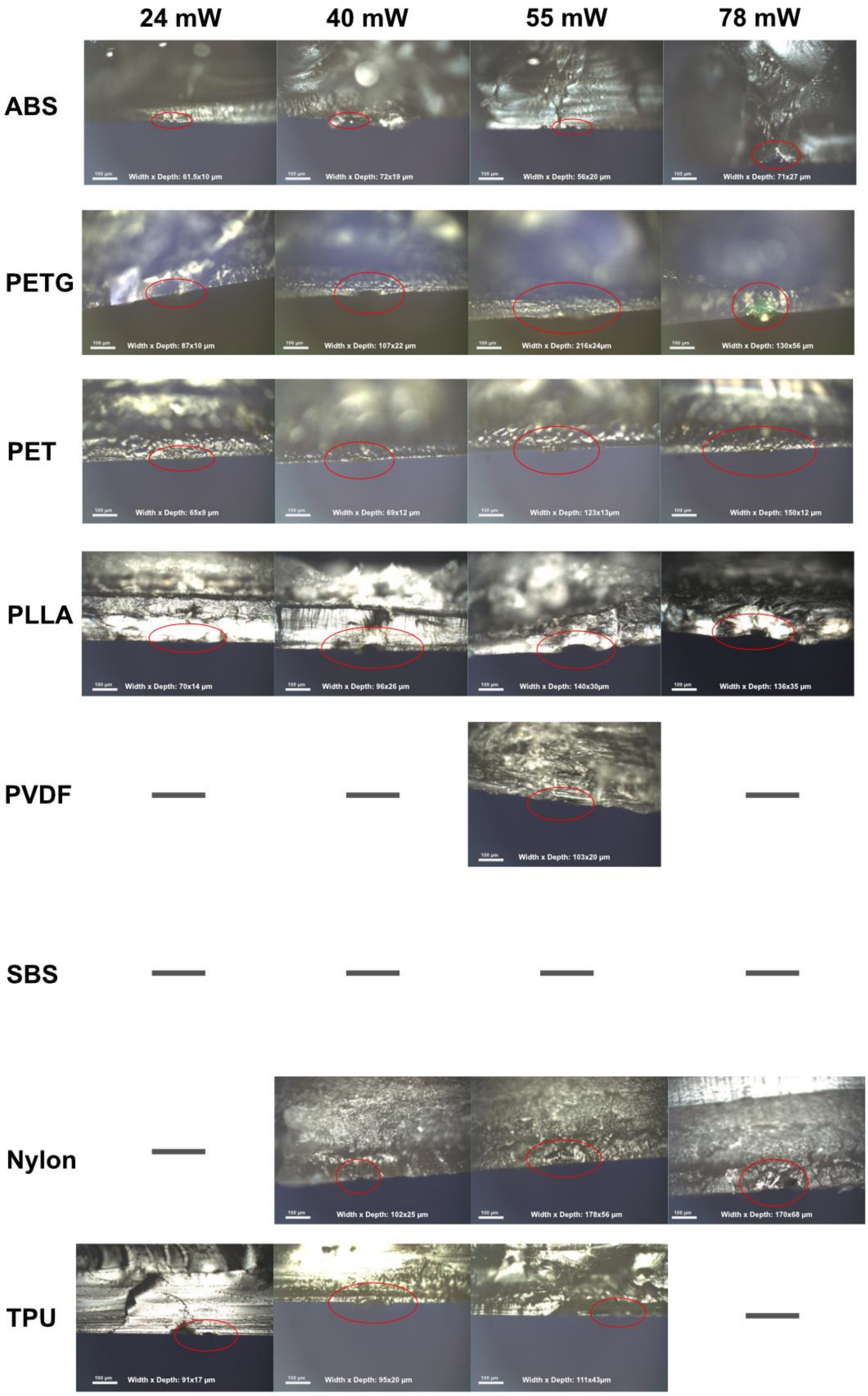

Figure S9. Cross-section optical microscopy of laser-induced lines at a power of 24, 40, 55, and 78 mW. Polymers were broken after 10 min in liquid nitrogen.

Table S4. Fe mass content (%) for the set of polymers processed with different laser power

Sample / Processing power	24 mW	40 mW	55 mW
TPU/rGO/Fe_3O_4	4.6	4.5	3.5
Nylon/rGO/Fe_3O_4	0.7	1.7	2.8
PVDF/rGO/Fe_3O_4	2.4	3.0	1.0
PETG/rGO/Fe_3O_4	3.9	3.9	3.4
PLLA/rGO/Fe_3O_4	0.7	1.6	N/A
SBS/rGO/Fe_3O_4	5.2	1.8	2.4
Reference (PVDF/GO/Fe_3O_4)	6.4		

References

1. Bucknall, C.B. *Toughened Plastics*; Applied Science Publishers, 1977; ISBN 9780853346951.
2. Dhotel, A.; Rijal, B.; Delbreilh, L.; Dargent, E.; Saiter, A. Combining Flash DSC, DSC and Broadband Dielectric Spectroscopy to Determine Fragility. *J. Therm. Anal. Calorim.* **2015**, *121*, 453–461.
3. Hohimer, C.; Christ, J.; Aliheidari, N.; Mo, C.; Ameli, A. 3D Printed Thermoplastic Polyurethane with Isotropic Material Properties. In Proceedings of the Behavior and Mechanics of Multifunctional Materials and Composites 2017; Goulbourne, N.C., Ed.; SPIE, April 11 2017.
4. Angell, C.A. Molecular Mobility in Polyurethane/styrene–acrylonitrile Blends Studied by Dielectric Techniques. *Eur. Polym. J.* **1999**, *35*, 923–937.
5. Peng, G.; Zhao, X.; Zhan, Z.; Ci, S.; Wang, Q.; Liang, Y.; Zhao, M. New Crystal Structure and Discharge Efficiency of Poly(vinylidene Fluoride-Hexafluoropropylene)/poly(methyl Methacrylate) Blend Films. *RSC Adv.* **2014**, *4*, 16849–16854.
6. SBS Rubber at a Glance Available online: https://pslc.ws/macrog/sbsg.htm (accessed on 17 October 2023).
7. Zhai, H.; Salomon, D. Evaluation of Low-Temperature Properties and the Fragility of Asphalt Binders with Non-Arrhenius Viscosity–temperature Dependence. *Transp. Res. Rec.* **2005**, *1901*, 44–51.
8. Guo, S.-L.; Chen, B.-L.; Durrani, S.A. Solid-State Nuclear Track Detectors. In *Handbook of Radioactivity Analysis*; Elsevier, 2020; pp. 307–407 ISBN 9780128143971.
9. Alaloul, W.S.; John, V.O.; Musarat, M.A. Mechanical and Thermal Properties of Interlocking Bricks Utilizing Wasted Polyethylene Terephthalate. *Int. J. Concr. Struct. Mater.* **2020**, *14*, doi:10.1186/s40069-020-00399-9.
10. Colwill, J.; Simeone, A.; Gould, O.; Woolley, E.; Mulvenna, C. Energy-Efficient Systems for the Sensing and Separation of Mixed Polymers. *Procedia CIRP* **2017**, *62*, 512–517.
11. Kováčová, M.; Kozakovičová, J.; Procházka, M.; Janigová, I.; Vysopal, M.; Černičková, I.; Krajčovič, J.; Špitalský, Z. Novel Hybrid PETG Composites for 3D Printing. *Appl. Sci.* **2020**, *10*, 3062.
12. Valvez, S.; Silva, A.P.; Reis, P.N.B. Optimization of Printing Parameters to Maximize the Mechanical Properties of 3D-Printed PETG-Based Parts. *Polymers* **2022**, *14*, doi:10.3390/polym14132564.
13. Soleyman, E.; Aberoumand, M.; Rahmatabadi, D.; Soltanmohammadi, K.; Ghasemi, I.; Baniassadi, M.; Abrinia, K.; Baghani, M. Assessment of Controllable Shape Transformation, Potential Applications, and

Tensile Shape Memory Properties of 3D Printed PETG. *J. Jpn. Res. Inst. Adv. Copper-Base Mater. Technol.* **2022**, *18*, 4201–4215.
14. La Gala, A.; Fiorio, R.; Ceretti, D.V.A.; Erkoç, M.; Cardon, L.; D'hooge, D.R. A Combined Experimental and Modeling Study for Pellet-Fed Extrusion-Based Additive Manufacturing to Evaluate the Impact of the Melting Efficiency. *Materials* **2021**, *14*, doi:10.3390/ma14195566.
15. Tarrazó-Serrano, D.; Castiñeira-Ibáñez, S.; Sánchez-Aparisi, E.; Uris, A.; Rubio, C. MRI Compatible Planar Material Acoustic Lenses. *Appl. Sci.* **2018**, *8*, 2634.
16. Clark, S.; Yap, T.; Tehrani, M. Validation of a Finite Element Model for Fused Filament Fabrication Additive Manufacturing. In Proceedings of the Volume 2A: Advanced Manufacturing; American Society of Mechanical Engineers, November 1 2021.
17. Flaata, T.; Michna, G.J.; Letcher, T. Thermal Conductivity Testing Apparatus for 3D Printed Materials. In Proceedings of the Volume 2: Heat Transfer Equipment; Heat Transfer in Multiphase Systems; Heat Transfer Under Extreme Conditions; Nanoscale Transport Phenomena; Theory and Fundamental Research in Heat Transfer; Thermophysical Properties; Transport Phenomena in Materials Processing and Manufacturing; American Society of Mechanical Engineers, July 9 2017.
18. Haleem, A.; Kumar, V.; Kumar, L. Mathematical Modelling & Pressure Drop Analysis of Fused Deposition Modelling Feed Wire. *Int. J. Eng. Technol.* **2017**, *9*, 2885–2894.
19. Spinelli, G.; Kotsilkova, R.; Ivanov, E.; Georgiev, V.; Naddeo, C.; Romano, V. Thermal and Dielectric Properties of 3D Printed Parts Based on Polylactic Acid Filled with Carbon Nanostructures. *Macromol. Symp.* **2022**, *405*, 2100244.
20. Xie, K.; He, Y.; Cai, J.; Hu, W. Thermal Conductivity of Nylon 46, Nylon 66 and Nylon 610 Characterized by Flash DSC Measurement. *Thermochim. Acta* **2020**, *683*, 178445.
21. Campanale, C.; Savino, I.; Pojar, I.; Massarelli, C.; Uricchio, V.F. A Practical Overview of Methodologies for Sampling and Analysis of Microplastics in Riverine Environments. *Sustain. Sci. Pract. Policy* **2020**, *12*, 6755.
22. Ngo, I.-L.; Jeon, S.; Byon, C. Thermal Conductivity of Transparent and Flexible Polymers Containing Fillers: A Literature Review. *Int. J. Heat Mass Transf.* **2016**, *98*, 219–226.
23. Choi, E.-Y.; Kim, K.; Kim, C.-K.; Kang, E. Reinforcement of Nylon 6,6/nylon 6,6 Grafted Nanodiamond Composites by in Situ Reactive Extrusion. *Sci. Rep.* **2016**, *6*, 37010.
24. Wang, C.; Hausberger, A.; Berer, M.; Pinter, G.; Grün, F.; Schwarz, T. Fretting Behavior of Thermoplastic Polyurethanes. *Lubricants* **2019**, *7*, 73.
25. Pinedo, B.; Hadfield, M.; Tzanakis, I.; Conte, M.; Anand, M. Thermal Analysis and Tribological Investigation on TPU and NBR Elastomers Applied to Sealing Applications. *Tribol. Int.* **2018**, *127*, 24–36.
26. Ameduri, B. From Vinylidene Fluoride (VDF) to the Applications of VDF-Containing Polymers and Copolymers: Recent Developments and Future Trends. *Chem. Rev.* **2009**, *109*, 6632–6686.
27. dos Santos, W.N.; Iguchi, C.Y.; Gregorio, R., Jr Thermal Properties of Poly(vinilidene Fluoride) in the Temperature Range from 25 to 210 °C. *Polym. Test.* **2008**, *27*, 204–208.
28. Phattaranawik, J.; Jiraratananon, R.; Fane, A.G. Heat Transport and Membrane Distillation Coefficients in Direct Contact Membrane Distillation. *J. Memb. Sci.* **2003**, *212*, 177–193.
29. Gradys, A.; Sajkiewicz, P.; Adamovsky, S.; Minakov, A.; Schick, C. Crystallization of Poly(vinylidene Fluoride) during Ultra-Fast Cooling. *Thermochim. Acta* **2007**, *461*, 153–157.
30. Kou, C.; Wu, X.; Xiao, P.; Liu, Y.; Wu, Z. Physical, Rheological, and Morphological Properties of Asphalt Reinforced by Basalt Fiber and Lignin Fiber. *Materials* **2020**, *13*, doi:10.3390/ma13112520.
31. Zheng, M.; Wu, S.; Wang, C.; Li, Y.; Ma, Z.; Peng, L. A Study on Evaluation and Application of Snowmelt Performance of Anti-Icing Asphalt Pavement. *Appl. Sci.* **2017**, *7*, 583.
32. Pan, P.; Wu, S.; Hu, X.; Liu, G.; Li, B. Effect of Material Composition and Environmental Condition on Thermal Characteristics of Conductive Asphalt Concrete. *Materials* **2017**, *10*, doi:10.3390/ma10030218.
33. Chen, J.; Li, L. Thermal Conductivity of Graphene Oxide: A Molecular Dynamics Study. *JETP Lett.* **2020**, *112*, 117–121.
34. Yang, Y.; Cao, J.; Wei, N.; Meng, D.; Wang, L.; Ren, G.; Yan, R.; Zhang, N. Thermal Conductivity of Defective Graphene Oxide: A Molecular Dynamic Study. *Molecules* **2019**, *24*, doi:10.3390/molecules24061103.
35. Zeng, Y.; Li, T.; Yao, Y.; Li, T.; Hu, L.; Marconnet, A. Thermally Conductive Reduced Graphene Oxide Thin Films for Extreme Temperature Sensors. *Adv. Funct. Mater.* **2019**, 1901388.
36. Mahanta, N.K.; Abramson, A.R. Thermal Conductivity of Graphene and Graphene Oxide Nanoplatelets. In Proceedings of the 13th InterSociety Conference on Thermal and Thermomechanical Phenomena in Electronic Systems; IEEE, May 2012.
37. Torrisi, L.; Cutroneo, M.; Torrisi, A.; Silipigni, L. Measurements on Five Characterizing Properties of Graphene Oxide and Reduced Graphene Oxide Foils. *Phys. Status Solidi* **2022**, *219*, 2100628.
38. Crica, L.E.; Dennison, T.J.; Guerini, E.A.; Kostarelos, K. A Method for the Measurement of Mass and Number of Graphene Oxide Sheets in Suspension Based on Non-Spherical Approximations. *2d Mater.* **2021**, *8*, 035044.

39. Zhang, H.; Fonseca, A.F.; Cho, K. Tailoring Thermal Transport Property of Graphene through Oxygen Functionalization. *J. Phys. Chem. C Nanomater. Interfaces* **2014**, *118*, 1436–1442.
